# Psychosocial training and support guidelines for research staff

**DOI:** 10.2471/BLT.21.287159

**Published:** 2022-07-13

**Authors:** Raquel Burgess, Andrew Wooyoung Kim, Lindile Cele, Seema Khadka, Kripa Sigdel, Ashley K Hagaman

**Affiliations:** a Department of Social and Behavioral Sciences, Yale School of Public Health, Yale University, 60 College St New Haven, Connecticut, 06510, United States of America (USA).; b Department of Anthropology, University of California, Berkeley, USA.; c Faculty of Health Sciences, University of the Witwatersrand, Johannesburg, South Africa.; d Helen Keller International Nepal, Lalitpur, Nepal.; e Department of Psychology, Tribhuvan University, Kathmandu, Nepal.

Over the past six decades, protections for the mental well-being of human participants in research have been included in national and international research ethics guidelines. Moreover, supporting mental health in the workplace has become an increasingly important focus in global public health given evidence of the role of job stress in the development of mental health issues.^1^ Despite efforts to protect the mental well-being of human research participants and the workforce in general, the research community has so far failed to provide recommendations to protect the mental health needs of the research workforce. Specifically, the research community has neglected to formally recognize that the research process can have important implications for the mental health of research staff, including research assistants, analysts and students.^2^^–^^6^ We have also overlooked the need to provide guidance for appropriate supports to minimize harm. These oversights may disproportionately impact research teams based in low- and middle-income countries because in many of these countries, large burdens of untreated mental health disorders exist and public mental health infrastructure and mental health-care provision has widespread limitations.^7^^,^^8^

Research, especially but not exclusively qualitative research, requires deep engagement with the topic matter, the lived experiences of participants, and the moral and political consequences of the findings. For many research projects, such involvement may mean significant emotional and intellectual effort is expended by research staff to engage with topics such as domestic violence, suicide and rape, which can lead to intense feelings of distress, shame, guilt, burnout, outrage and hopelessness.^2^^–^^6^ Moreover, this distress may be dealt with using negative coping strategies such as emotional numbing, avoidance or substance abuse.^3^^,^^5^^,^^9^ The emotional burden of research is likely to be heavier for individuals who are a member of the community that they are studying.^4^^,^^5^ Some research staff, including those working in local communities, may experience magnified risk as the result of ongoing societal adversities including classism, racism and sexism. Other relevant stressors include personal safety risks while working in the field^6^ and pressure to complete work quickly and to a high standard. One of our co-authors, a research assistant on a study based in South Africa, shared:

“I know I often felt ill-prepared in such situations and I dealt with it by becoming emotionally numb but felt guilty and shameful for being so, but it was the only way I knew how to cope because the work had to be done, targets needed to be met. Thus, I placed my own humanity – or morality or ability to connect, feel, relate to someone – on the backburner in favour of the work that needed to be done.”

Despite the mental health risks associated with the job responsibilities of research staff, the broader cultural and economic contexts in which many global health studies are based may prevent them from speaking about their distress. In many low- and middle-income countries, the low availability of mental health-care services is compounded by high levels of mental health stigma. In Nepal, for example, the limited mental health workforce and tightly connected social network of psychosocial workers can make it challenging to maintain confidentiality. 

In many regions of the world, these challenges are exacerbated by the lack of basic employee benefits and unsupportive workplace conditions experienced by research staff in fieldwork and research settings. The temporary and transient nature of employment contracts in research studies can preclude employees from accessing crucial health-care benefits such as trauma counselling and long-term psychotherapy, limit their opportunities to develop relevant skills, and create a sense of prolonged job insecurity. All these factors combined may stunt career progression. Lead investigators often oversee global health studies remotely and depend on local staff to coordinate daily research operations without direct supervision, which may prevent investigators from implementing the necessary mental health protections to shield study staff from poor working conditions, limited benefits and other occupational hazards. Researchers have begun to highlight the educational and career disparities that result from unsupportive work environments and power imbalances in various aspects of global health research, including unequal access to training and publishing opportunities as well as barriers to gaining research independence.^10^ These entrenched disparities are well-known predictors of lower job satisfaction, decreased productivity and adverse mental health outcomes.^11^^,^^12^ Another co-author based in Nepal describes: “High demand of job obligations, insufficient salary and benefits, poor working conditions, and the lack of operational guidance on mental health of the research staff in the countries like Nepal pose a mental health risk.”

Professionals in clinical fields and practice-based professions such as psychology, social work and health care have recognized and responded to these risks by developing supports such as distress prevention toolkits to prevent clinicians from experiencing vicarious trauma, post-traumatic stress disorder, depression and/or anxiety.^12^ However, despite the multifaceted mental health risks faced by research staff, little guidance is available to help investigators recognize and understand these risks and provide appropriate supports. The authors’ review of resources provided by international and North American institutions such as the World Health Organization (WHO), the National Institutes of Health, the American Public Health Association, the American Anthropological Association, the American Psychological Association and others reveals a paucity of practical guidelines for protecting the mental health of research staff. Our review of several professional agencies in south Asia and South Africa (including, for example, the Nepalese Psychological Association and the Health Professions Council of South Africa), revealed the same findings. Additionally, our personal experiences conducting trauma and mental health research in global settings, as well as various discussions with our colleagues also working in adverse environments, reveal that best practices are primarily cultivated through the passing on of operational wisdom from more experienced mentors and partner organizations.

The passing down of operational wisdom in this context is valuable, but insufficient. This informal knowledge sharing creates too much dependence on the less experienced investigator to have the foresight, time and access to the expertise of more experienced investigators to adequately protect the well-being of the larger research team. We call upon the academic community and organizations such as WHO and regional professional associations to fund the development of a comprehensive and accessible set of guidelines to assist investigators in protecting the mental health needs of their research staff. Moreover, the protection of research staff should be enshrined in the review processes of research ethics boards and funders and in the culture of academic institutions.^9^^,^^13^ Additional research and higher quality data are required to better understand the mental health demands that research staff face, and which supports are most effective in alleviating these demands. Using the findings from our literature review and our collective experience, we have aggregated recommended supports and identified entities responsible for these supports throughout each phase of the research process (Box 1).

Box 1 **Stages of research, responsible entities and ways to support mental health of researchers**

**Pre-preparation phase**

Investigators, institutions and grant agencies are responsible for investigator training that includes:

- Identifying mental health demands of the proposed research
- Identifying available mental health resources and gaps in their availability
- Identifying alternative support systems where resources are not available (for example, telehealth, non-profits willing to provide support)
- Structuring regular staff engagement with mental health resources
- Monitoring use of resources
- Establishing a culture of mutual respect and support
- Implementing techniques and strategies to provide regular debriefing with research staff
- Recognizing and responding to distress (for example, through psychological health first aid)

**Preparation phase**

Investigators are responsible for identifying and planning for specific mental health needs that includes:

- Identifying mental health demands of the research
- Communicating clearly to prospective staff about mental health risks;^2^^,^^6^ requiring “informed consent” from staff
- Identifying staff mental health needs (fears, triggers, forms of acceptable psychosocial resources)
- Planning to minimize the potential for research to exacerbate pre-existing mental health issues (for example, allow staff to structure their workload in a way that optimizes their mental health)
- Planning for periodic in-person check-ins at research site, to identify risks and challenges experienced by staff

Investigators, institutions, research ethics boards and grant agencies are responsible for research staff training that includes:

- Recognizing and responding to personal and colleague distress (psychological health first aid)
- Engaging in self-reflection to identify own sources of fear, triggers, signs of burnout and forms of acceptable psychosocial resources
- Keeping a journal to identify and process distressing emotions and thoughts
- Developing techniques for practicing self-compassion and self-care^3^^,^^5^^,^^6^
- Developing strategies for engaging in emotional distancing and boundary setting when needed^2^^,^^3^
- Developing an understanding of professional and personal limits with respect to assisting participants^4^
- Learning about trauma-informed practice (research teams gain knowledge and understanding of trauma and its far-reaching implications)
- Learning about psychoeducation (basic knowledge of mental distress and crisis management)
- Developing basic emotional coping skills (mindfulness)
- Understanding and engaging referral processes for mental health resources and care
- Developing strategies for effective use of mental health resources
- Providing refresher trainings through the course of the project, especially as new risks arise and study conditions change

Investigator, research ethics boards and grant agencies are responsible for maximizing impact of the research findings by:

- Conducting planning to ensure the project has a positive impact outside of the academic context^2^^,^^6^ to validate the study staff’s contributions and maximize the study’s broader social and public health effects. Research staff who are members of the community being studied are meaningful participants of this process

**Data collection and analysis phase**

Investigators, institutions, research ethics boards and grant agencies are responsible for recognizing research staff’s contribution by:

- Providing opportunities for staff to be recognized for the difficult work they are doing (authorship, opportunities to present at conferences)^10^

Investigators and research ethics boards are responsible for facilitating transition out-of-role by:

- Holding debriefing session(s) to allow staff to process their experiences

**Throughout the research process**

Investigators and research ethics boards are responsible for understanding and supporting staff with intersectional realities by:

- Developing an awareness of the intersectional realities (classism, racism, sexism) that many staff face and ensuring that this awareness is applied to understand psychosocial needs. Awareness may be achieved through historical review of the research context, literature reviews, extensive experiential fieldwork and ethnographic research. Staff are empowered to assist with the planning of the research study to address these realities and have regular opportunities to discuss any challenges they may be facing with the investigator and research ethics board as needed

Investigators are responsible for self-reflection and behaviour by:

- Acting as role models to the staff (for example, taking breaks), being aware of the implicit or explicit expectations placed on staff and modifying expectations in accordance with the mental health risks of the research (for example, providing flexible timelines for work completion)^7^

Note: Although we believe that these supports should be provided as a minimum requirement in almost all research projects, not all the recommended supports may apply to every research study in every context. Moreover, the investigator is encouraged to consider additional supports that may be needed to support their research staff given the demands of their specific research study.

In the past two years, the coronavirus disease 2019 pandemic has increased the exposure of most of the world’s population to psychosocial stressors.^14^ These effects have been felt most intensely by those in low- and middle-income settings, those with insecure employment and those who were already dealing with other psychosocial stressors.^15^^,^^16^ Now is a critical time to prioritize the development of resources to support the mental health of research staff, with the aim of ensuring that all participants of the research process are sufficiently protected and prepared for the mental health demands of emotionally challenging research. If, in the pursuit of public health, we value the development of evidence base on complex societal problems, we must similarly value the need to support our research staff to empower, affirm and embrace their own mental health needs.
